# Cochlear implantation in patients with inner ear schwannomas: a systematic review and meta-analysis of audiological outcomes

**DOI:** 10.1007/s00405-024-08818-3

**Published:** 2024-07-11

**Authors:** Francesco P. Iannacone, Torsten Rahne, Elisabetta Zanoletti, Stefan K. Plontke

**Affiliations:** 1 Department of Neuroscience DNS, Otolaryngology Section, University of Padova, Padua, Italy; 2 Department of Otorhinolaryngology, Head and Neck Surgery, University Medicine Halle, Martin Luther University Halle-Wittenberg, Ernst-Grube-Str. 40, 06120 Halle (Saale), Germany

**Keywords:** Acoustic neuroma, Cochlear implant, Inner ear schwannoma, Intracochlear, Intralabyrinthine, Neurotology, Schwannoma, Skull base surgery, Vestibular schwannoma

## Abstract

**Purpose:**

In patients with inner ear schwannomas (IES), reports on hearing rehabilitation with cochlear implants (CI) have increased over the past decade, most of which are case reports or small case series. The aim of this study is to systematically review the reported hearing results with CI in patients with IES considering the different audiologic outcome measures used in different countries.

**Methods:**

According to the Preferred Reporting Items for Systematic Reviews and Meta-Analyses (PRISMA) guideline, a search of published literature was conducted. We included patients with IES (primary or with secondary extension from the internal auditory canal (IAC) to the inner ear, sporadic or NF2 related) undergoing cochlear implantation with or without tumour removal. The audiological results were divided into the categories “monosyllables”, “disyllables”, “multisyllabic words or numbers”, and “sentences”.

**Results:**

Predefined audiological outcome measures were available from 110 patients and 111 ears in 27 reports. The mean recognition scores for monosyllabic words with CI were 55% (SD: 24), for bisyllabic words 61% (SD: 36), for multisyllabic words and numbers 87% (SD: 25), and 71% (SD: 30) for sentences. Results from for multisyllabic words and numbers in general showed a tendency towards a ceiling effect. Possible risk factors for performance below average were higher complexity tumours (inner ear plus IAC/CPA), NF2, CI without tumour removal (“CI through tumour”), and sequential cochlear implantation after tumour removal (staged surgery).

**Conclusion:**

Hearing loss in patients with inner ear schwannomas can be successfully rehabilitated with CI with above average speech performance in most cases. Cochlear implantation thus represents a valuable option for hearing rehabilitation also in patients with IES while at the same time maintaining the possibility of MRI follow-up. Further studies should investigate possible risk factors for poor performance. Audiological tests and outcome parameters should be reported in detail and ideally be harmonized to allow better comparison between languages.

## Introduction

Cochleovestibular schwannomas are benign tumours arising from the Schwann cells anywhere along the course of the eighth cranial nerve [1]. When such tumours arise within the bony labyrinth, they are termed intralabyrinthine schwannomas or, more precisely, inner ear schwannomas (IES). Due to technological advancement in diagnostic tools and greater awareness of this condition in the last two to three decades, the incidence rate of IES has risen to 1.1 per 100,000 person-years reaching 4.1 for individuals with age of 70 years and above [2]. Most IES patients initially present with hearing loss and at the time of therapy show non-serviceable hearing and tinnitus [3–6]. Management strategies of these tumours include observation, radiotherapy, or surgery [6]. Initially, common indications for surgery were growth towards the cerebellopontine angle (CPA) or middle ear, intractable vestibular symptoms, or to clarify the pathologic diagnosis [3, 7]. Hearing rehabilitation in these patients can be achieved either through transferring sound reaching the affected ear to the contralateral ear with hearing aids or bone-anchored hearing systems (Contralateral Routing of Signal: CROS) or through cochlear implantation (CI) of the affected ear. Cochlear implantation in patients with single sided deafness or asymmetric hearing loss leads to improved sound localization, hearing in noise, and to significant improvements in quality of life, particularly in subjects with associated tinnitus [8–10]. In patients with IES, reports on hearing rehabilitation with cochlear implants have increased over the past decade, most of which, however, are case reports or small case series. The aim of this study is to systematically review the reported audiologic outcomes after cochlear implantation in patients with IES considering different audiologic outcome measures used in different countries.

## Materials and methods

### Search method

According to the Preferred Reporting Items for Systematic Reviews and Meta-Analyses (PRISMA) guideline [11], a search of published literature on Pubmed, Scopus and OVID databases was conducted. The last search was performed on April 13, 2024. The following research strings with relevant keywords and Mesh terms were used: “(("Neurilemmoma"[Mesh]) AND ((intralabyrinthine) OR (intracochlear) OR (intravestibular))) AND (("Cochlear Implantation"[Mesh]) OR ("Cochlear Implants"[Mesh]))”; “(TITLE-ABS-KEY (*neuroma) AND TITLE-ABS-KEY ( *intralabyrinthine OR *intracochlear OR *intravestibular)) AND TITLE-ABS-KEY ("Cochlear Implantation" OR "Cochlear AND Implants")”; “Intracochlear schwannoma OR intravestibular Schwannoma OR intralabyrinthine schwannoma AND cochlear implant”. The results were exported to Zotero bibliography manager (v6.0.30, Center for History and New Media, George Mason University, Fairfax, Virginia) to remove duplicates and then screened for eligibility. Additionally, the references of the selected articles were screened for possible further publications that could be considered for inclusion.

Inclusion criteria were: (1) patients with inner ear schwannomas (primary IES or with secondary extension from the IAC to the inner ear) undergoing cochlear implantation with or without tumour removal; (2) availability of single patient data (sex, age at surgery, laterality, but especially for: tumour localization, treatment modality, pre and post-operative audiological outcomes, whether NF2-related schwannomatosis was present as underlying disease or not). Exclusion criteria were: (1) language of publication other than English, Spanish or Italian; (2) lack of specification of the speech audiometry test used or, alternatively, lack of specification of speech material used, and (3) time point of speech audiometry test result not reported. From the selected studies, data on demographics, patient/tumour characteristics and audiological outcome were extracted and documented in a data base. The resulting studies were fully reviewed and the following studies were excluded: studies without information on tumour localization [12], without outcome data for individual patients [13, 14], and without specified time-points of audiological examination(s) [15–19]. The remaining studies were analysed for updated reports of the same patients and only the most recent patient and audiological data were used for analysis [20–27]. Only published data were used for the analyses.

### Subgroup analysis

The audiological results were divided into the categories “monosyllables”, “disyllables”, “multisyllabic words or numbers”, and “sentences”. A summary of the audiometric speech tests used, and their main characteristics is shown in Table 1. When multiple audiological outcomes at different time-points after cochlear implantation for a specific patient were published, only the latest available time point after implantation was considered for the analysis.

**Table 1:** Demographics, patient and tumour characteristics, and speech audiometry tests reported in the selected studies.

Patient characteristics (N = 110)	
*Sex (N = 110)*	*N (%)*
Male	56 (51)
Female	46 (41)
Not specified	9 (8)
*Laterality (N = 111)*^a^	*N (%)*
Right side	45 (40)
Left side	46 (41)
Not specified	20 (19)
*Mean age at CI surgery (range) (N* = *95)*	50.2 years (23–83 y)
*NF2 status (N = 110)*	*N (%)*
No	86 (78)
Yes	24 (22)
*Tumour localization (N = 111)*	*N (%)*
IC	60 (54)
IV	17 (15)
IVC	7 (6)
IE + IAC/CPA	27 (25)
*Tumour management (N = 111)*	*N (%)*
Single surgery with CI	85 (77)
Staged surgeries	5 (5)
Radiotherapy^b^	8 (7)
Observation (tumour in situ)	13 (11)
*Speech audiometry tests description*	
*Words*	
- CNC (consonant-nucleus-consonant) [69]	- 500 monosyllabic words organized in lists of 50 words, equally phonemic distribution. RV. Recommended PL 70 dB SPL
- Fournier lists [70]	- Six lists of 50 bisyllabic phonemically balanced; often half-lists used. Adaptive test
- Turrini-Cotugno lists[71]	- Lists of 10 bisyllabic phonemically balanced words. Adaptive test
- Freiburg monosyllables and numbers tests [72]	- Lists of monosyllabic words and lists of 10 numbers. Fixed PL (usually 65 dB SPL)
*Sentences*	
- HINT (Hearing in Noise Test) [69]	- 250 sentences derived from Banford-Kowal-Bench organized in 25 lists of 10 sentences. RV. Recommended PL 70 dB SPL
- CUNY (City University of New York) sentences [73]	- 48 lists of 12 sentences comprising 102 words, topic-related sentences. Fixed PL (usually 65 dB SPL)
- BKB (Bench-Kowal-Bamford Sentence) [74]	- 20 lists of 16 short sentences with contextual information. Fixed PL (usually 70 dB SPL)
- Hochmaier–Schulz–Moser (HSM) Test [75]	- 30 lists of 20 everyday sentences. RV. Fixed PL (usually 60-, 65-, or 75-dB SPL)
- AzBio (Arizona Biomedical Sentences) [56]	- 15 lists of 20 sentences with 3–12 words with limited contextual cues. RV. Fixed PL (usually 60 dB SPL)

Percentages are provided with respect to tumours (ears) or patients, as appropriate

*CI* cochlear implant, *NF2* neurofibromatosis type 2, *IC* intracochlear, *IV* inntravestibular, *IVC* intravestibulocochlear, *IE* inner ear, *IAC* internal auditory canal, *CPA* cerebellopontine angle, *PL* presentation level, *SPL* sound pressure level, *RV* recorded voice

^a^One bilateral case from Quick et al. [47]

^b^One case of radiotherapy for head ependymoma (patient 5 from Eitutis et al. [33])

Tumour location was classified as intracochlear IES (IC), intravestibular IES (IV), intravestibulocochlear IES (IVC), and IES with extension through the fundus (transfundal) to the internal auditory canal or even to the cerebellopontine angle (IES + IAC/CPA) according to a revised classification on IES [28] which was modified after Kennedy et al. [3] and Van Abel et al. [5]).

The following subgroups were analysed: (1) “*sporadic IES or NF2-related IES*”; (2) “*CI with tumour removal or CI without tumour removal, and *(3) “*IC, IV, IVC, or IE* + *IAC/CPA*”. Intracochlear tumours can be removed with various degrees of opening the cochlea ranging from extended cochleostomy, or partial or subtotal cochlectomy to push-through/pull-through techniques [23, 29–31]. In cases, where cochlear implantation was done without tumour removal, this meant that the CI was inserted into the cochlea despite or through the tumour [32–34].

Speech recognition at one year of CI usage was categorized based on an international outcome analysis of a large cohort [35]. Monosyllabic and disyllabic word recognition scores below 50% at conversational level, which is close to the reported mean word recognition score (WRS) with CI across languages were categorized as “below average” (“poor performers”). Based on those patients where both, monosyllabic and sentence recognition scores were available, we determined the sentence recognition score equivalent to a WRS of 50% using a nonlinear regression with Pade (1,1), which showed that sentence recognition scores below 85% could be categorized as “below average” (R-squared: 0.75).

### Statistics

Statistical analyses were carried out using Prism Version 10, GraphPad, San Diego, USA. Descriptive statistics were calculated for all variables of interest, and data are presented as mean and standard deviation. Mean recognition scores for monosyllables and sentences were compared between the subgroups of *sporadic IES* vs. *NF2-related IES* and *CI with tumour removal* vs. *CI through tumour* using unpaired two-tailed t-tests. Due to the small sample sizes, recognition scores for disyllables as well as multisyllabic words and numbers were reported descriptively only. The effect of tumour location on the mean recognition scores was assessed by one-way analyses of variances (ANOVA) with the between-subject factor of tumour location (*IC, IV, IVC, IES* + *IAC/CPA*). Alpha was set to 0.05 for all tests.

## Results

### Patient and treatment characteristics

**Fig. 1:**
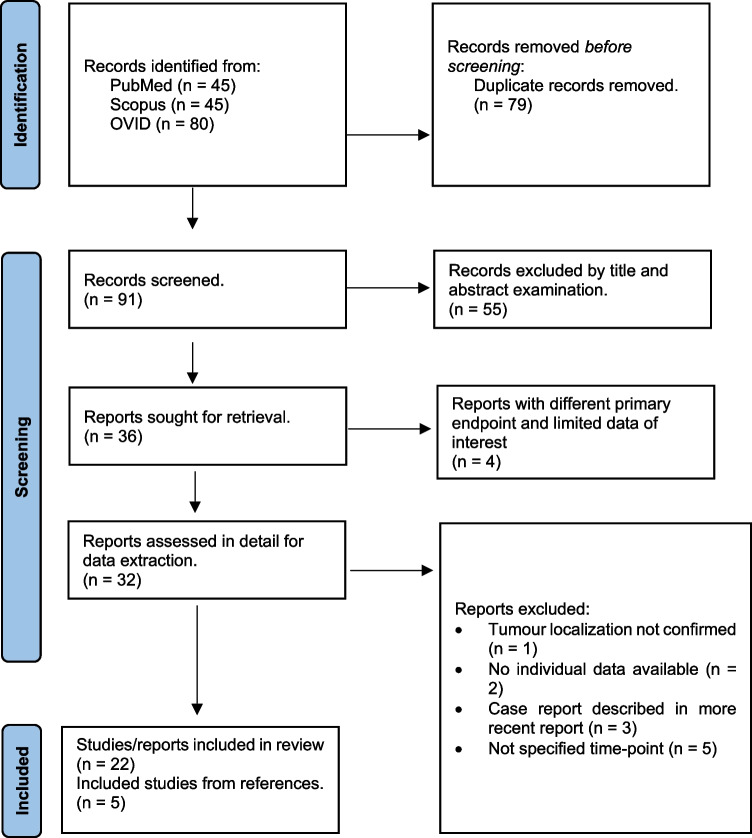
PRISMA flow chart

Twenty-seven papers fulfilled the inclusion criteria (see PRISMA Flow chart in Fig. 1), for a total of 169 patients with IES reported [20, 22, 23, 25, 26, 29, 30, 32–34, 36–53]. After exclusion of two patients with placement of a dummy electrode, two patients with failure to preserve the modiolus, seven patients treated without CI and 25 duplicate/updated reports, 133 patients were used for the analysis. The predefined audiological outcome measures were available from 110 patients, for a total of 111 measurements (one patient with bilateral IES). The demographic details of those patients are summarized in Table 1. Eighty-five patients were treated with surgical removal of the tumour. Different surgical techniques were used, and the surgical steps were described in varying degrees of detail. Surgical management of intracochlear IES was mainly based on two groups of approaches. The first (n = 27) consisted in “pulling out” the tumour mass through an enlarged round window, through a single or double cochleostomy or through drilling of the bony wall of the basal turn. The second (n = 31) involved more extensive drilling of the cochlear capsule and removal of the tumour through a partial or subtotal cochlectomy. This technique was also used for IES with transfundal extension to the IAC and the CPA (n = 3) and in one patient with unilateral, multifocal schwannoma of the inner ear and the CPA. Intravestibular IES were resected through a labyrinthectomy or—in case of growth from the vestibule to the IAC (without involvement of the modiolus)—through a translabyrinthine approach. Intravestibulocochlear IES with or without growth to the IAC where resected through a combination of the approaches described above. Cochlear implantation with simultaneous tumour removal (n = 84) was the most common form of treatment in terms of timing of hearing rehabilitation. Two patients were implanted sequentially after tumour removal [29]. Twenty-one patients were implanted after worsening of hearing loss during observation or after radiotherapy [32–34, 51]. Five patients with NF2-related schwannomatosis underwent multiple surgeries due to the presence of tumour in the cerebellopontine angle. The mean last audiological follow-up was 18.6 months (1–180, SD: 21).

### Audiological outcomes

**Fig. 2:**
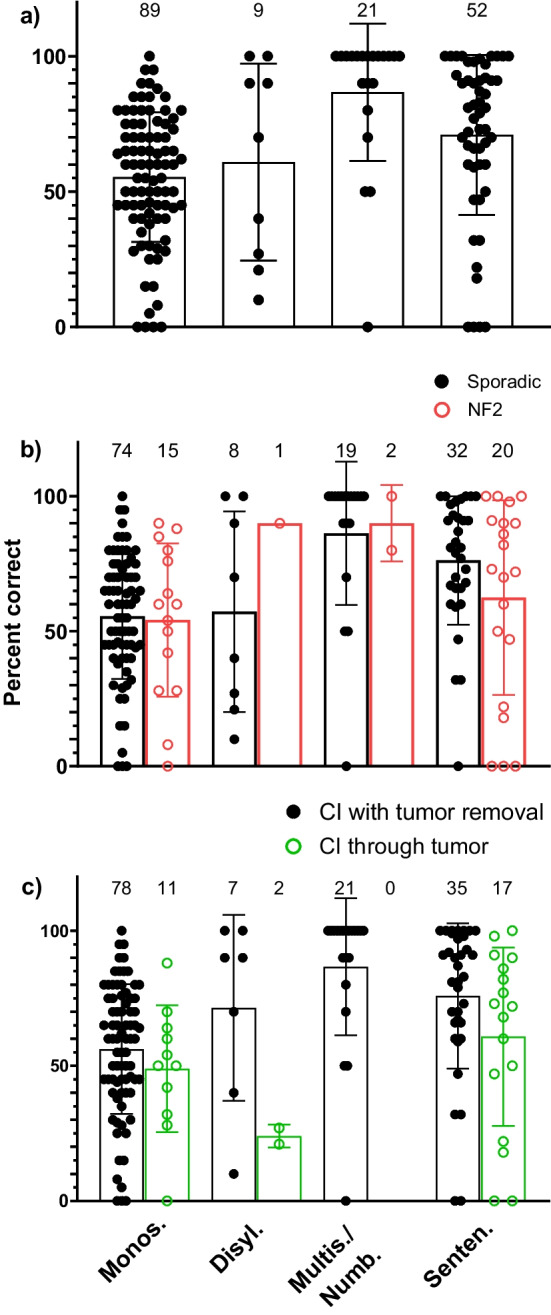
Audiological outcomes with various speech material (mean and standard deviations): **a** Entire patient cohort (n = 110 patients and 111 ears), **b** sporadic IES and NF2-related IES; **c** tumours treated with microsurgical tumour removal and simultaneous or sequential CI and patients with cochlear implantation and tumour left in situ (“CI through tumour”), i.e., with observation or after radiotherapy*.* The number of available data are shown above the columns. Outcome measurements for different speech audiometry tests may belong to the same patient. A tendency for better CI performance in patients with sporadic IES (sentences) and for CI with tumour removal (monosyllables and sentences) but no statistically significant difference between the groups was found (*t* tests, all *p*s > 0.05). The time point of measurement is not considered here, which may introduce bias (compare Fig. 3). *CI* cochlear implant, *NF2* Neurofibromatosis type 2 related schwannomatosis. *Monos.* Monosyllables, *Disyl.* disyllabic words, *Multis./Numb.* multisyllabic words or numbers. *Senten.* sentences

**Fig. 3:**
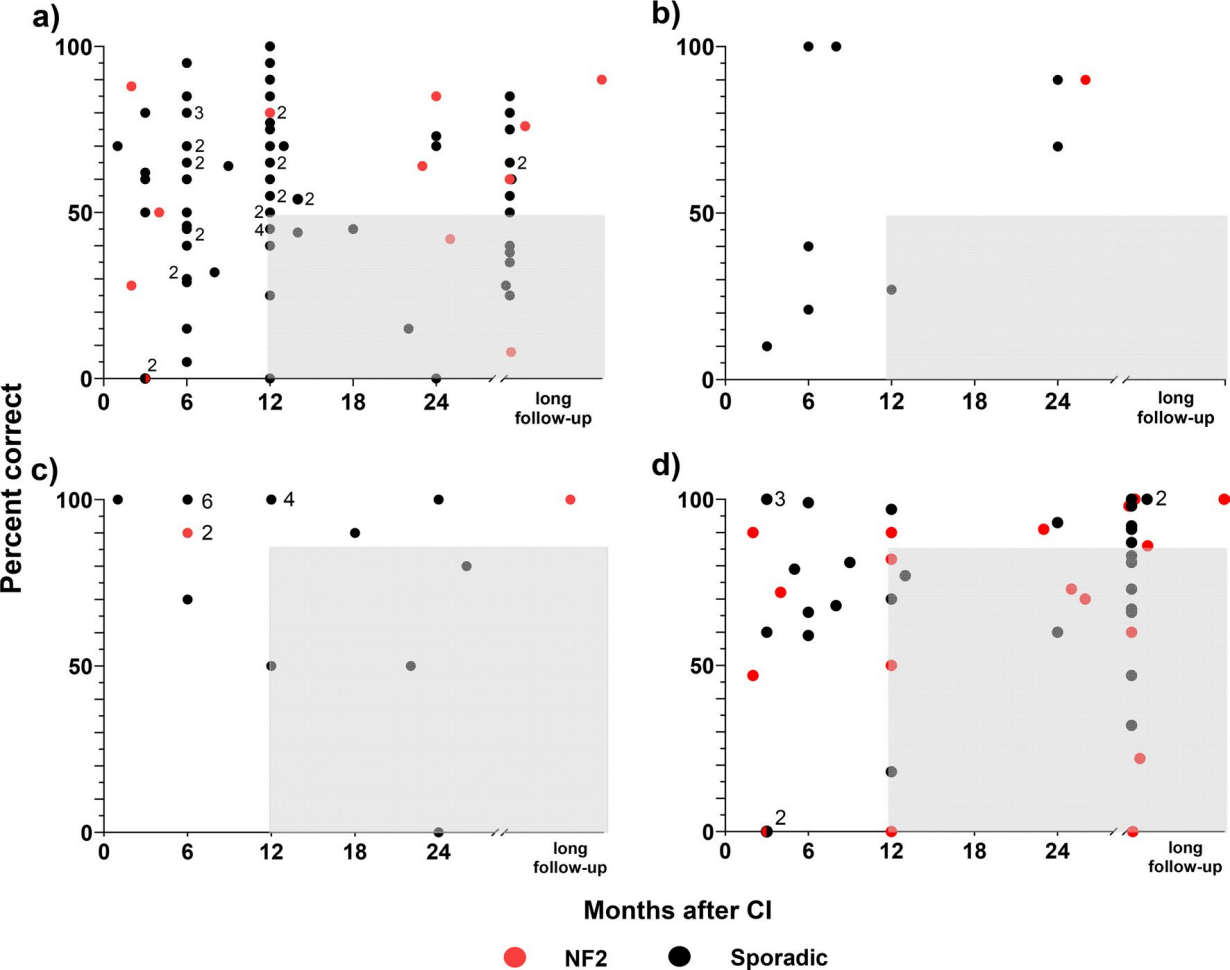
Individual audiological outcomes of included population: **a** speech audiometry outcomes using monosyllabic word lists (n = 89), **b** speech audiometry outcomes using disyllabic word lists (n = 9), **c** speech audiometry outcomes using multisyllabic words and multisyllabic numbers (n = 21), **d** speech audiometry outcomes using sentences in quiet (n = 52)*.* The grey area shows the "poor performers”, i.e., recognition scores of < 50% for monosyllabic and disyllabic tests or < 85% for multisyllabic words or numbers and sentences in quiet, respectively, after at least 12 months follow up (for definition: see methods). Numbers next to data points indicates multiple patients (ears) with the same outcome. *CI* cochlear implant, *NF2* Neurofibromatosis type 2 related schwannomatosis

**Fig. 4:**
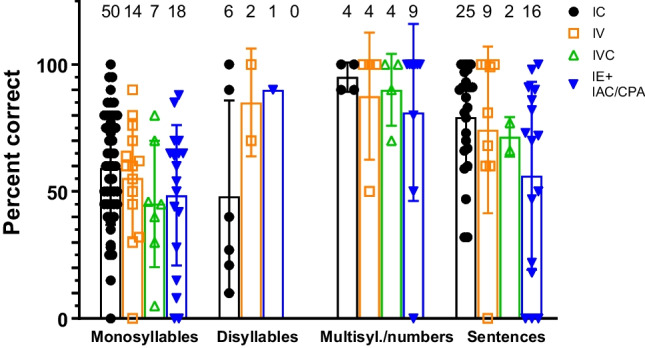
Audiological outcomes based on tumour localization (mean with standard deviation): The number of available data are shown above the columns. Outcome measurements for different speech audiometry tests may belong to the same patient. A tendency for better CI performance in patients with intracochlear IES but no statistically significant effect of tumour location on mean recognition scores was observed (ANOVA, all *p*s > 0.05). The time point of measurement is not considered here, which may introduce bias (compare Fig. 3). *IC* intracochlear inner ear schwannoma (IES), *IV* intravestibular IES; *IVC* intravestibulococlhear IES; *IE + IAC/CPA* IES with primary or secondary involvement of internal auditory canal and/or cerebellopontine angle

Audiological outcomes for individual patients in the entire patient cohort and for the predefined subgroups are shown in Figs. 2, 3 and 4. The graphs include outcomes for different speech audiometry tests of the same patient if tested with multiple speech materials (e.g., monosyllables and numbers, or monosyllables and sentences). Eighty-nine ears were tested with monosyllables, nine with disyllabic, twenty-one with multisyllabic words or numbers, and fifty-two with sentences in quiet. For monosyllabic and disyllabic outcome measures the following test were used: Freiburg monosyllabic test (n = 57), consonant-nucleus-consonant test (CNC) (n = 22), Mandarin and Korean monosyllabic test (n = 5), Cutugno-Prosser-Turrini test (n = 4), Fournier disyllables test (n = 3) and unspecified tests (n = 7). The tests were performed at presentation levels of 65 dB SPL (n = 85), 60 dB SPL (n = 4), 70 dB SPL (n = 2) and at unspecified levels (n = 7). For multisyllabic and sentence outcome measures the following test were used: Freiburg numbers test (n = 20), Hochmaier–Schulz–Moser (HSM) Test in quiet (n = 15), AzBio (n: 16), Bench–Kowal–Bamford (BKB) sentence test (n = 7), City University of New York (CUNY) sentence lists (n = 4), Fournier sentence test (n = 3), Mandarin and Korean sentences (n = 2), Hearing in Noise Test (HINT used in quiet) (n = 1), and unspecified tests (n = 5) presented at 65 dB SPL (n = 16), 60 dB SPL (n = 16), 70 dB SPL (n = 9), and at unspecified levels (n = 32).

The mean recognition scores for monosyllabic words were 55% (SD: 24), for disyllabic words 61% (SD: 36), for multisyllabic words and numbers 87% (SD: 25), and 71% (SD: 30) for sentences (Figs. 2a, 3).

In the subgroup “*sporadic IES or NF2-related IES*” the mean recognition scores were 56% (SD: 23) and 54% (SD: 28) for monosyllables; 57% (SD: 37) and 90% for disyllables, 86% (SD: 27) and 90% (SD: 14) for multisyllabic words and numbers, and 76% (SD: 24) and 62% (SD: 36) for sentences in quiet, respectively (Fig. 2b). A tendency for better CI performance (sentences) in patients with sporadic IES but no statistically significant difference for mean monosyllabic and sentence recognition scores between the *sporadic IES* and *NF-related IES* subgroups was found (all *p*s > 0.05).

In the subgroup “*CI with tumour removal or CI through tumour”,* the mean recognition scores were 56% (SD: 24) and 49% (SD: 26) for monosyllables, 71% (SD: 34) and 24.0% (SD: 4.2) for disyllables, 87% (SD: 25) for multisyllabic words and numbers (CI through tumour: n = 0), and 76% (SD: 27) and 61% (SD: 33) for sentences in quiet, respectively (Fig. 2c). A tendency for better CI performance in patients with tumour removal and CI but no statistically significant difference for the mean recognitions scores for monosyllables and sentences between the *CI with tumour removal* and *CI without tumour removal* subgroups were found (all *p*s > 0.05).

In the subgroup *“IC, IV, IVC, IES* + *IAC/CPA”* means of correctly understood monosyllables were 59% (SD: 22), 55% (SD: 23), 45% (SD: 25), and 49% (SD: 28); for disyllables 48% (SD: 38), 85% (SD: 21), and 90% (no results available from IV); for multisyllabic words and numbers 95% (SD: 5.8), 88% (SD: 25), 90% (SD: 14), and 81% (SD: 35, and for sentences in quiet; 79% (SD: 21), 74% (SD: 33), 72% (SD: 7.8), 56% (SD: 37), respectively (Fig. 4). A tendency for better CI performance in patients with intracochlear IES but no statistically significant effect of tumour location on mean recognition scores for monosyllables (*F*(3,85) = 1.36, *p* > 0.05), disyllables (*F*(2,6) = 1.19, *p* > 0.05), multisyllabic words and numbers (*F*(3,17) = 0.28, *p* > 0.05), and sentences (*F*(3,48) = 2.15, *p* > 0.05) was found.

## Discussion

### Test materials and CI performance

The results from this systematic review study show that hearing loss in most patients with IES can be successfully rehabilitated with cochlear implants. The mean audiological outcomes (55% for monosyllables and 71% for sentences) are similar to those of hearing rehabilitation with CI for several other aetiologies of hearing loss. In a recent review based on a large group of CI patients, the mean performance for monosyllables was 54% (SD: 23) and 74% (SD: 37) for sentences in quiet across different speech materials [54]. As can expected, monosyllabic and disyllabic words tests showed lower recognition scores then using multisyllabic words and numbers and sentences in quiet (Fig. 2a–c). Results for multisyllabic words and numbers in general showed a tendency towards a ceiling effect, which was also found in the subgroups ‘sporadic IES’, ‘CI with tumour removal’ and for IC, IV, or IVC IES, respectively (Figs. 2b, c, 3c, 4). Tests using sentences in quiet are known for their ceiling effect, especially at later measurement time points after cochlear implantation. Gifford et al. [57] showed that 28% of the subjects tested with sentences in quiet (HINT sentence material used in quiet) achieved a maximum performance of 100% and that scores did not agree well with monosyllables or sentence recognition in noise. In the cohort presented in this systemic review, 23 patients (44%) scored > 85% at sentences in quiet (AzBio, CUNY, BKB, and HINT material used in quiet), and, when tested also with monosyllabic, their results varied from 38 to 90%. It should be noted that the presentation levels in these studies, in which the stimulus level was reported, varied in a range between 60 and 70 dB SPL. However, variation in stimulation levels in this range may only have a small effect on the results [55]. Speech in noise tests can overcome disadvantages of ceiling effects in less sensitive speech audiometry tests [56, 57]. Only a few articles reported speech recognition in noise [30, 34, 37, 43, 50, 52, 53]. In these articles, good performances during tests in noise are reported, in some cases reaching a level close to normal-hearing ears.

### Influence of NF2

Cochlear implantation in NF2-related IES was described by various authors [32, 37, 44, 46]. In our analysis, similar speech recognition scores were found in sporadic and NF2-related IES for all speech material except for sentences, where a tendency for a lower performance of NF2 patients was observed (Fig. 2b). Schwannomas in the inner ear in NF2-patients usually present as secondary growth from the IAC to the inner ear. Primary IES in NF2-related schwannomatosis appears to be extremely rare. Jia et al. [37] described a primary IES in an NF2 patient and mentioned that only three cases in their database of more than 250 NF2-patients presented with a primary IES. Of note is also the first described case of cochlear implantation after sequential bilateral microsurgical resection of intracochlear IES in a patient without NF-related schwannomatosis and good audiological outcomes (77% for monosyllables and 97% for sentences at the first side and 73% for monosyllables and 93% for sentences for the contralateral ear after CI) [47, 58].

### Tumour extent and CI performance

Intracochlear and intravestibular IES showed similar outcomes, although the surgical trauma of the cochlea is clearly larger after removal of tumours from the cochlea. Patients with tumours filling both, the cochlear and vestibular parts of the inner ear (intravestibulocochlear IES) or with higher complexity tumour extensions (inner ear and IAC/CPA) showed a tendency towards poorer monosyllabic and sentence recognition scores and a higher percentage of “poor performers” (Fig. 4). Nevertheless, hearing rehabilitation with CI seems possible in the short to medium term, even if the tumour extends from the inner ear to the IAC. With respect to MRI follow-up, ipsilateral inner ear, IAC and CPA can be adequately visualized for tumour surveillance after CI [32, 37, 46, 59, 60]. The most recent models of CI systems are all approved for MRI at 1.5 T and 3 T by using rotating magnets in the receiver coil. In order to increase the chance of acceptable visibility of the areas of interest, i.e., the inner ear and the IAC, some measures should be considered. A comparison of the artifact size obtained from 1.5 and 3 T MRIs for three different systems (Advanced Bionics 3D, MED-EL Synchrony and Oticon ZTI) showed no major differences in terms of maximum artifact sizes [61]. Temporary magnet removal for MRI can also significantly reduce the artifacts. For assessing the IAC and the labyrinth, using a 2D MRI sequence is suggested instead of a 3D MRI sequence (Drive, CISS) [62]. Images from 2D-T2 and 2D-T1 with gadolinium offer similar visibility of the inner ear [59, 62]. The nonmagnetic components of a CI electrode produce a local “cancellation” effect on the fluid signal in T2-weighted sequences, which however that does not prevent the detection of an IES [63], allowing potential growth of tumour remnants or recurrences to be identified. Chin-to-chest position in the MRI scanner enhances the visualization of the IAC and cochlea especially in the coronal plane [64]. Finally, the CI receiver coil position should be customized to allow for surveillance of further tumourigenesis in other encephalic areas [65].

### Surgical tumour removal versus CI through tumour

The first cochlear implantation in a patient with a confirmed IES in the basal turn of cochlea was published in 1999 [16]. Two years earlier Tono et al. described a case of a 48-years old patient with an intracanalicular schwannoma with apparent positive enhancement within the vestibule indicating possible transmacular extension of the tumour. After removal of only the intracanalicular tumour parts via a middle fossa approach and no further exploration of the vestibule, the patient received a CI fifteen months after tumour surgery reaching recognition scores of 60% and 81% for monosyllabic and sentences in quiet, respectively 1 year after implantation [12]. A similar case was described by Ahsan et al. in 2003, however, the authors did not have access to formal speech audiometry tests in Spanish, and therefore, these tests were not performed [15]. It is only in the last decade that an increasing number of patients have been treated with surgical removal of IES and simultaneous or sequential cochlear implantation, including cases of single sided deafness [10, 17–20, 22–26, 29, 30, 37, 39, 43–48]. On the one hand, this trend could be related to the increasing evidence of the benefits of cochlear implantation in terms of hearing rehabilitation, sound localization, decrease of tinnitus-related complaints and overall quality of life in SSD patients [8–10, 13]. On the other hand, it could be due the good audiological results and the possibility of preservation of vestibular receptor function even in cases where significant resection at the cochlear capsule is necessary to remove the tumour [20, 39]. Patients treated with tumour removal (including a large number with partial or subtotal cochlectomy) showed a tendency towards better performances with CI compared to those where the tumour was left in situ (“CI through tumour”). This effect could be attributed to better approximation of the electrode to the modiolus with the spiral ganglion cells in Rosenthal’s canal and to better isolation compared to a regular, perilymph filled cochlea with the consequence of reduced spread of electric field [66].

### Risk factors for performance below average

Inclusion of early follow up data may introduce bias towards poor performance. When applying the above-mentioned criteria for “poor performance” (below average with respect to CI in general; grey areas in Fig. 3) to those patients with follow-up of at least 12 months, a total of 44 measurements were below average (Fig. 3). Among the 17 patients with intracochlear IES and below average results, three scored 45% [20, 40], two scored 40% [48], one scored 38% [52] and five scored below 35% [29, 40, 48, 51] with monosyllabic tests, one scored 27% with a disyllabic test [34], six scored between 83% and 66% and three scored under 50% with sentences test in quiet [41]. For the patients with sporadic IES, scores below 30% for monosyllables and disyllables were found in higher complexity tumours (inner ear plus IAC/CPA) [26], in patients with “CI through tumour” [34] and in patient with sequential cochlear implantation after tumour removal (staged surgery) [29]. The high rate of suboptimal performers in the NF2 cohort (n = 10; 42%) was likely related to various reasons. These patients suffered from high complexity IES at the time of surgery (inner ear plus IAC/CPA), decline of CI performance in patients with tumour recurrence in the IAC, or radiotherapy followed by cochlear implantation without tumour removal. Therefore, long term outcome in patients with sporadic IES is expected to be better than in NF2-related schwannomatosis. If tumours are only partially resected, this also inherently involves a risk for future tumour growth and potential decline in performance. As several risk factors appear to be concentrated in the NF2 cohort, the significance of each factor for the final audiologic outcome is not yet clear.

Recent studies showed that performance of ‘regular’ CI users can be predicted based on duration of deafness, age, and maximum preoperative word recognition [67]. Thus, success of cochlear implantation should rather be judged on an individual basis. Since many patients with IES, e.g., have a short duration of deafness, the model would predict rather good speech perception results. We assume that IES as a cause of hearing loss and the type of surgery will add additional predictive factors to the model and should be developed in the future.

### Agreements and disagreements with other reviews and meta-analysis

Only one review (a scoping review) has been published on this topic so far [68]. In that review, the authors compared the audiometric performance level, CI user status and open-set speech scores between patients with IES with surgical removal and tumour in situ, categorizing outcome in performance classes. Some of the included studies were excluded from our study for not meeting the inclusion criteria [12, 15, 16, 18]. Conversely, we included patient outcomes from a number of studies that were not considered by Wang et al. [20, 22, 23, 26, 38]. A statistical analysis was not performed by the authors due to data heterogeneity.

### Limitations of the study

There are some limitations of our study. The study design of included reports (mostly case reports and retrospective case series) represents a limiting factor for proper statistical analysis. Publication bias may also have influenced the overall outcomes analysis. Pooling speech recognition outcomes across languages is challenging and methods for this are not standardized. In many studies the speech audiometry specifications (e.g., speech level, speech material, number of items, masking procedures, live voice, or recorded voice) have not been reported adequately, thus introducing bias. Speech tests based on sensation level would also be misleading in CI users, since the speech reception threshold is mainly determined by the fitting parameters. Correction factors between speech tests are mainly based on the intelligibility function which is not available for CI users. Further, CI users may use different cues of the speech material than people with unaided acoustic hearing do. Further studies should thus report sufficient information about the speech materials used to decrease the exclusion rate in such studies with a rare condition. An alternative would be the use of speech tests in noise that are available in many languages. Language specific correction factors could then be derived as differences to the language specific normative data.

## Conclusions

Speech performance of CI users after IES surgery is above average in most cases and represents a valuable option for hearing rehabilitation in those patients. Further studies should investigate possible risk factors for poor performance (e.g., tumour extension [inner ear plus IAC/CPA], NF2, “CI through tumour”, staged surgery, previous radiotherapy). More medium and long-term follow-up date are of importance since reporting of early follow-up data only, likely introduces bias. Audiological tests and outcome parameters should be reported in detail and ideally be harmonized to allow better comparison between languages.

## Data Availability

Not applicable.
